# A Mental Hospital Sketch

**Published:** 1914-04-18

**Authors:** 


### A Mental Hospital Sketch.

### THE IDIOT.

I wonder how many of us there are who, upon being told that a certain institution was at one and the same time a home, a hospital, and a boarding-school for idiots, would have either the courage or the interest to investigate for himself. But were such a visit to attract him, how amply would he be repaid! I had met a few idiots previously, certified and otherwise, but until quite recently I had never seen them in their proper place.

So far as the Eastern Counties are concerned, this is at Colchester, adjoining the station. It is one of those places which suggest the acquirement of a country mansion and estate, ruined for that purpose by the proximity of the railway, but which, by judicious alterations and additions, admirably serves the purpose to which it has been put. The day of my visit was overshadowed, even in the midst of Christmas festivities and prize-giving, by the death of the Father of the Institution, the man who, above all others, had made the institution what it is, and had secured for its 300 patients, by voluntary support, that home, that hospital care, and that boarding-school, which otherwise could only have been met by residence in a county or city institution, perhaps to the detriment of their fellow-patients. By judicious gradation and segregation each child is placed in the grade where he will be of the least hindrance to the progress of others, and uninfluenced detrimentally by those with whom he is classed. Thus, setting aside those requiring treatment in the hospital wards, and those for whom nothing can be done apart from ordinary nursemaid care as to guarding them from harm, and attending to their cleanliness, there remains that other grade who can be, and are, usefully employed. For these there is the schoolroom, where the children—for they are that throughout life; they never "grow up" as we understand it—are taught something of the things which go to make up their surroundings, and their poor minds are developed somewhat. Later they are employed in the making and repairing of clothes, mats, brushes, and boots; farm work, weaving, laundrying, house work, and outdoor games. But best of all is their woodwork, both cabinet-making and carving; some of their chip carving and relief-work will take its place amongst the best examples of that art. The patients are kept in a state of cleanliness, and taken such care of as would be impossible in their own homes; they are well fed, and whenever possible they are usefully occupied. Such an institution as this has no room for those who merely seek employment in the hope of receiving some monetary reward, from the medical officer to the humblest employee in charge of patients all work with that love and devotion which characterises the institutional worker, whether in this country or abroad. In an institution such as this, however, what surprises even a fellow institutional worker is that very atmosphere of love and devotion. Amongst those in the "Hospital" one expects it; amongst those even in the "School" and workrooms one is not altogether surprised to find it, because *some* result is obtained—and in certain cases a very fine result—whether as a bandsman or in any of the other branches of industry to which reference has already been made. But it is in the "Home" and amongst the low grade of patient that there exists in its highest degree that spirit of devotion manifesting itself in the constant care of those for whom nothing—positively nothing—can be done apart from what, for want of more apt description, one might call nursemaid care.

